# Prevention of child mental health problems through parenting interventions in Southeastern Europe (RISE): study protocol for a multi-site randomised controlled trial

**DOI:** 10.1186/s13063-021-05817-1

**Published:** 2021-12-27

**Authors:** Diana Tăut, Adriana Băban, Inga Frantz, Ingrid Dănilă, Jamie M. Lachman, Nina Heinrichs, Catherine L. Ward, Frances Gardner, Xiangming Fang, Judy Hutchings, Marija Raleva, Galina Lesco, Hugh Murphy, Heather Foran

**Affiliations:** 1 Department of Psychology, Babeș-Bolyai University, Cluj-Napoca, Romania; 2 Department of Psychology, Clinical Psychology, and Psychotherapy, University of Bremen, Bremen, Germany; 3 Centre for Evidence-Based Intervention, Department of Social Policy and Intervention, University of Oxford, Oxford, UK; 4 MRC/CSO Social and Public Health Sciences Unit, University of Glasgow, Glasgow, UK; 5 Department of Psychology, University of Cape Town, Cape Town, South Africa; 6 School of Public Health, Georgia State University, Atlanta, GA USA; 7 School of Psychology, Bangor University, Bangor, UK; 8 Institute for Marriage, Family and Systemic Practice – ALTERNATIVA, Skopje, North Macedonia; 9 Health for Youth Association, Chișinău, Republic of Moldova; 10 Institute for Psychology, University of Klagenfurt, Klagenfurt, Austria

**Keywords:** Child behaviour problems, Parenting, RCT, Parent training, LMIC

## Abstract

**Background:**

Childhood adversities, such as poor parental practices, exposure to violence, and risk behaviours strongly impact children’s future mental and behavioural problems. Adversities affect families living in disadvantaged environments and low- and middle-income countries (LMICs) to a greater extent than in high-income countries. Parenting programmes are an effective way to alleviate them, although their outreach and scalability is still limited in LMICs.

**Methods/design:**

A multi-site randomised controlled trial will be conducted in North Macedonia, Republic of Moldova and Romania to test the efficacy and cost-effectiveness of an optimised version of the promising Parenting for Lifelong Health Programme for Young Children (PLH-YC, 5 sessions), against a standard lecture on parenting issues (control group, 1 session). At least 864 participants who report having children between 2 and 9 years old who display elevated levels of behavioural difficulties will be randomised on a 1:1 basis to the intervention and control groups. The primary outcome will consist of parent report of child oppositional aggressive behaviour. Post-test (four months) and follow-up (12 months) assessments will provide information on short- and longer-term effects of PLH-YC compared to the parenting lecture in the control group.

**Discussion:**

This randomised trial will test the efficacy of PLH-YC in alleviating child behavioural problems and assess the cost-effectiveness, transportability across three different cultural contexts, and potential for scalability of the programme.

**Trial registration:**

ClinicalTrials.gov., Registration number: NCT04721730 (https://clinicaltrials.gov/ct2/show/NCT04721730). Registered 13.01.2021

**Supplementary Information:**

The online version contains supplementary material available at 10.1186/s13063-021-05817-1.

## Background

The health of children and adolescents plays a central role in the public health agenda with salutary results: morbidity and mortality from communicable diseases have steadily decreased since 1990 [1]. However, the World Health Organization (WHO) cautioned that, despite being preventable, youth mental health problems continue to be overlooked, leaving youth unable to achieve their full potential as adults [2]. Globally, between 10 and 20% of children suffer from mental health problems, which account for an estimated 16% of the global burden of disease and injury in those aged 10–19 years [3]. Much of the burden associated with impaired mental health is carried by young people in low- and middle-income countries (LMICs), who make up roughly 85% of the total child and adolescent population in the world [4]. In the European Region, the proportion of adolescents from LMICs who struggle with emotional difficulties is generally higher than of those facing the same difficulties in high-income countries (HIC). For instance, almost 33% of the Romanian, 26% of the Moldovan, and 19% of the North Macedonian adolescents, aged 11 to 15, reported feeling low more than once a week, compared to 13% on average across the 45 countries included in the survey [5].

Socio-economic disadvantages and child mental health tend to intersect, through exposure to immediate adversity that impacts on parenting, caregivers’ own mental health and relational issues, and their risk behaviours, increasing the risk of child abuse [6–9]. The Adverse Childhood Experiences Study (ACE) [10] conducted in eight LMICs illustrated this with 30% of Moldovan, 27% of Romanian, and 21% of North Macedonian children subjected to physical abuse compared to 4–16% in HICs [11]. In addition to exposure to physical abuse, 22% of children in Romania witness someone in the household having an alcohol problem and 14% live with a depressed or suicidal household member. In North Macedonia, emotional neglect (17%), followed by problematic alcohol use (9%), and depression/suicidal ideation in one household member (7%) were the top three most frequently mentioned ACEs. Exposure to multiple adversities has exponential effects on future adults: youngsters exposed to physical abuse are more likely to start smoking early, experiment with high-risk drugs, run away from home, attempt suicide, or engage in sexual risk behaviours [12–15].

Despite challenges, many children who grow up living with adversity become healthy adults. The protective role of an emotionally warm and competent caring system in helping children to overcome adversities and in fostering social and emotional regulation skills is well-established [16]. Parenting in disadvantaged communities, however, is fraught with difficulties that undermine both parents’ own mental health and their ability to provide protective parenting. In addition, the exposure of children to community adversity that increases child behaviour problems is, in itself, a factor that may elicit harsh, inconsistent parental practices [17–19]. Parenting programmes are key to assisting caregivers who raise families affected by adversities and have been shown to be effective in improving both parental practices [20, 21] and reducing child maltreatment and children’s emotional and behavioural problems [21–23]. Some programme components appear particularly effective in reducing disruptive behaviours in children: positive reinforcement, praising desired behaviours, and applying natural or logical consequences [24]. These components are shared across many different evidence-based parenting programmes, having their roots in social learning theory and the programme design approach of Hanf [25]. However, they are usually costly and difficult to transfer to, and scale-up in, LMICs [26]. The risk in rolling out such programmes is that their cost may prevent them from reaching the most disadvantaged individuals and communities, thus potentially widening the social inequality gap by being accessible mostly to more well-off families and communities [27]. This would represent a huge missed opportunity. There is encouraging evidence that the components of these interventions can be transposed and successfully adapted to LMICs provided that the cultural particularities of these countries are taken into consideration whilst retaining their fundamental core principles [28, 29]. Strong involvement of local communities is also key to both successful adaptation and scale-up [30].

The existing evidence base highlights opportunities to address the mental health problems of children in LMICs, but also warns about milestones that need to be reached. There are still relatively few rigorously conducted randomised controlled trials (RCTs) of structured parenting programmes in LMICs [31], and it is unclear whether or how the accumulation of adversity interacts with programme components in enhancing or hampering beneficial effects. There are even fewer data on whether, and how, these interventions work across different contexts and in different LMICs, and whether they are similarly cost-effective [29]. Multi-site RCTs, like the present one, to be conducted in North Macedonia, Republic of Moldova and Romania, provide an opportunity to address these important questions and provide high-quality data to support local decision-making and service consolidation.

### Overview and aims of the present study

The present study is part of a cross-country project financed by the European Commission (Project Number 779318), called “Prevention of Child Mental Health Problems in Southeastern Europe - Adapt, Optimise, Test, and Extend Parenting for Lifelong Health (RISE).” The programme tested in this project is an adaptation of the Parenting for Lifelong Health for Young Children (PLH-YC) programme, for parents of children between 2 and 9 years old. PLH is a joint initiative of the World Health Organization, UNICEF, Clowns Without Borders South Africa, and a number of universities around the world, targeting families of infants, children, and adolescents to reduce violence against children and improve child wellbeing. It focuses on consolidating parenting skills involved in relationship building (spending one-to-one time with children and emotional coaching), positive reinforcement of children’s adaptive behaviours (praising and rewarding, providing positive instructions, setting household rules, and routines) and teaching positive discipline strategies (ignoring negative attention seeking and unreasonable demands, time-out, and establishing reasonable consequences for inappropriate behaviours) [32]. The programme is designed to be accessible and easy to integrate into communities’ existing childcare or social services and requires no particular professional background, although a PLH training programme for facilitators is strongly encouraged. PLH was tested in LMIC with promising results in South Africa [33, 34] and the Philippines [35].

The RISE project uses the Multiphase Optimisation Strategy (MOST) framework to optimise the intervention by taking into consideration effectiveness, cost-effectiveness, and scalability [36] in these three LMICs. The project aim is threefold: Phase 1 (Preparation) to implement a systematic empirical process in order to adapt contents and materials of PLH-YC for use in the three LMICs (surface adaptation) and test their feasibility in a small pre-post study; Phase 2 (Optimisation) to test the efficacy and cost-effectiveness of different programme components in order to select the most effective and cost-effective components for an optimised intervention; Phase 3 (Evaluation) to test the optimised intervention (identified in Phase 2) in an RCT conducted in the three LMICs, taking into consideration the broader socio-economical, cultural, and contextual factors relevant within, and across, the three countries. The protocols for the first two phases (Preparation and Optimisation) were presented in detail elsewhere [32, 37].

The present protocol draws on the third objective of RISE, as part of the Evaluation phase of MOST, by describing in detail the multi-site RCT.

The primary aims and subsequent trial hypotheses are to:

1. Test the effectiveness of the optimised PLH-YC programme in comparison to a parenting lecture in a multi-site RCT in three Southeastern European countries: North Macedonia, Republic of Moldova and Romania on the *primary outcome* child oppositional aggressive behaviour assessed by parent report of the level of child aggressive behaviour, prevalence of child externalising disorders (oppositional defiant disorder and conduct disorder—ODD/CD), and daily reports of child oppositional and aggressive behaviour. We hypothesise that (a) child aggressive behaviour and prevalence of child externalising disorders will be significantly reduced at the post-assessment in the PLH-YC programme condition compared to the control condition; (b) these effects will be maintained over the long term (pre-intervention to follow-up change, post-intervention to follow-up stability of change); (c) past 24-h reports of child oppositional and aggressive behaviour as measured repeatedly between pre- and post-assessment will significantly decrease over the months of assessments in the PLH-YC condition compared to the control group.
2. Test the effectiveness of the PLH-YC programme versus a parenting lecture on *secondary outcomes*, namely reductions in child internalising problems, dysfunctional parenting, child maltreatment, and parenting stress, as well as in improvements in daily reports of effective parenting behaviour, positive parenting, quality of parent-child relationship, parental mental health, and child quality of life. Based on the results of the previous phases, we also expect increases in parental relationship quality and decreases in intimate partner violence (physical and psychological victimisation and perpetration) in the parents’ relationship. We hypothesise that (a) there will be significant improvements in secondary outcomes at post-assessment to the advantage of PLH-YC; (b) these effects to be maintained at the follow-up assessment (pre-intervention to follow-up change, and post-intervention to follow-up stability of change); and that (c) past 24-h reports of effective parenting behaviours will significantly improve over the weeks of assessment in the PLH-YC programme condition compared to the control condition.

If the optimised programme is effective, a secondary objective of the RISE project is to explore the implementation and scalability of PLH-YC to enable wide-spread and sustained use of the optimised parenting programme in the Southeastern European countries. We will apply the steps of the RE-AIM model [38] to translate the learning from implementation of the evidence-based programme into sustainable practice. Moreover, we will report on the cost-effectiveness of the programme and other service use by parents in the PLH-YC and control conditions during, and after the intervention across the three countries.

The secondary aims and hypotheses are:

1. Examine the cost-effectiveness of PLH-YC in comparison to a lecture on parenting on the *primary outcome* of child aggressive behaviour and the economic impact of the programme, including potential costs for future dissemination and scale-up. Impact on other outcomes such as dysfunctional parenting, positive parenting, and child quality of life will also be used to better inform decision-making.
2. To assess the role of socio-economic, contextual and individual factors (i.e. family adversity, parental mental health, intimate partner violence, couple dissatisfaction, and child aggressive behaviour at baseline) on the implementation of PLH-YC in the three countries. We will examine implementation in hypotheses a–c in two ways. We will examine these associations with enrolment (attended 1st session) across groups. We will also examine the associations with participation rate in the PLH-YC condition (percentage of sessions attended). The hypotheses are:
  - Higher family adversity at baseline assessment—including higher poverty, household hunger, parental mental health problems, intimate partner violence, and couple dissatisfaction will be associated with reduced participation in the PLH-YC group and lower enrolment across groups;
  - Higher child aggressive behaviour at baseline will be associated with increased participation and enrolment by parents;
  - Higher programme participation and enrolment will be associated with greater improvements in the primary and secondary outcomes;
  - Higher programme fidelity and quality of delivery by facilitators of PLH-YC will be associated with greater improvements in the primary and secondary outcomes.
  - We also hypothesise that indirect effects such that the above listed baseline variables (a and b) will predict enrolment, participation rate, and programme fidelity, which in turn will predict improvements in primary and secondary outcomes.
3. *Additional Moderation Analyses*: Based on previous literature [27, 39, 40], we advance additional moderator hypotheses that pertain to parental, family, and child characteristics. Regarding parent/family characteristics, the hypotheses are as follows: (a) where parents have higher levels of baseline mental health problems, intervention effects on child aggressive behaviour will be greater in the PLH-YC compared to the control condition; (b) where parents have higher levels of baseline dysfunctional parenting, intervention effects on child aggressive behaviour will be greater for the PLH-YC condition compared to the control condition; (c) there will be no moderation effect of higher poverty, household hunger, and parental relationship dissatisfaction on the primary outcome child aggressive behaviour. The hypotheses related to child factors as moderators of intervention effect on the primary outcome are the following: (a) where children have higher levels of baseline child aggressive behaviour, intervention effects on aggressiveness will be greater, and (b) there will be no moderation effect of child gender or age on the primary outcome child aggressive behaviour (although gender may moderate outcome if associated with baseline level of child aggressive behaviour).

## Methods

### Study design

We aim to recruit 864 primary caregivers of children aged 2 to 9 years old from North Macedonia, Romania (both classified as upper middle-income countries; the World Bank categorised Romania as high-income country based on 2019 per capita income for the first time) and Republic of Moldova (lower middle-income country; *n* = 288 per country). Participants will be randomly allocated to one of the two parallel study arms: control group (consisting of a lecture) or a parenting intervention group receiving a five-session PLH-YC programme.

#### Recruitment

Recruitment of participants and baseline assessments are scheduled to take place from December 2020 to February 2021 in all three countries, a timeline which loosely corresponds to the middle of the 2020/2021 school year. However, the COVID-19 pandemic might cause some variation as study teams follow national policies and timelines regarding the new school year and access to schools.

Potential participants will be referred to the study by school counsellors, teachers and educators, or community workers. Additionally, all countries will advertise and provide study information on relevant websites and social media pages. On first contact with the research team, parents will be assessed for eligibility, and scheduled for providing informed consent and pre-assessment data. After the baseline assessment, they will be randomly allocated to one of the study conditions. During the recruitment stage, we will encourage secondary caregivers (i.e. fathers, grandparents, social tutors) to enrol for assessment and to take part in the programme, alongside primary caregivers.

### Participants and eligibility criteria

Potential parents or other primary caregivers will have the following inclusion criteria: (a) be aged 18 years or older, (b) be responsible for the care of a child between the ages of 2 and 9; (c) report at least subclinical levels of child’s behavioural problems as assessed with the oppositional defiant disorder subscale (ODD) of the Child and Adolescent Behavior Inventory (CABI, scores ≥ 10 will be included) [41, 42]; (d) have spent at least four nights a week with the child in the same household during the previous month and will continue to do; (e) agree to being randomised to one of the conditions; (f) consent to participate in the full study; (g) have adequate language skills to participate in the group /lecture, either in the primary language of the group or with additional language support provided. We will exclude primary caregivers whose children have been removed from their custody.

Inclusion criteria for facilitators are as follows: 1) aged 18 or older, 2) prior participation in a training workshop for the lecture/PLH-YC, 3) agreement to either deliver the lecture (one session) or PLH-YC (five sessions), 4) provision of consent to participate in the full study.

#### Group allocation and allocation concealment

Prior to randomisation, all countries will identify and select a sample of recruitment sites (schools, kindergartens, community, and social services centres) covering different neighbourhoods, geographical areas, ethnic compositions, and socio-economic strata in and around Skopje (North Macedonia), Cluj-Napoca (Romania), and Chișinău (Republic of Moldova).

Randomisation will be coordinated by one of the project partners (University of Klagenfurt) using an online randomiser, https://www.randomizer.org/ (*N* = 288 per country, 864 in total). Participant numbers will be randomised to the PLH-YC programme or the control condition after completion of the baseline assessment based on the randomisation list (individual 1:1 randomisation in blocks of 24). The randomisation list will be provided to the team in Bremen, who will be contacted after each participant is enrolled to unveil their assignment. This threefold procedure allows the analysis team to remain blind to group assignment, ensures that assignment occurs independently and ensures that teams in each country receive allocation information after enrolment.

Participants will be allocated to groups after completion of the pre-assessment. Thus, outcome assessors and participants will be blinded to group allocation at pre-assessment. All data assessors conducting pre-test, periodic, post-test, and follow-up interviews with parents will be blinded regarding the allocation of participants to the groups to minimise evaluation bias: The implementation of the intervention will be conducted by different staff (programme coordinators and facilitators) than the assessments. Thus, data assessors will be unaware of the group allocation of participants at the later assessment points (periodic, post, follow-up). To ensure that participants do not reveal their group allocation during the post- and follow-up assessment to the assessor, the data assessor will ask the participants to not share the group allocation at the beginning of the interview. Only in cases where the parent reports a (serious) adverse event, will the group allocation be unblinded (during periodic  and follow-up assessments). Upon completion of the pre-test measurements, caregivers will be notified of their allocation status.

Although one-to-one randomisation will be used, this will done in blocks of 24 (12 control and 12 PLH-YC) and vary in implementation for Republic of Moldova compared to Romania and North Macedonia. In the Republic of Moldova, participants within each of the 12 recruitment sites will be randomly allocated to either the control or the intervention due to the geographical spread of the sites across the country. In Romania and North Macedonia, participants will be assigned to any PLH-YC programme or any control lecture group and not based on region, but rather based on scheduling availability.

### Procedure

The interviews will be conducted in-person or over the telephone by trained data assessors, with experience in conducting field research with semi-structured interviews. In order to be assessed and further take part in the study, primary and consenting secondary caregivers will give verbal or signed consents to data assessors. If the interviews are conducted over the phone, the data assesors will send the *Information Sheet and the Informed Consent* (Appendix 1) to the participant ahead of time. During the interview, they will go over the Information Sheet with the participant and then present the informed consent. If the participant agrees verbally with each item from the informed consent, the data assessors will fill in the respective form, write the participant’s name and sign it with the data assessors’ name. For in-person interviews, the same procedure will apply, but the participant will fill in and sign the informed consent with their own names.

Data from the measurement points (pre-test, periodic, post-test, and follow-up) will be collected by using Open Data Kit (ODK), installed on individual tablets. This software was used in the previous two study phases and proved to be very well accepted and easy to adapt to the languages of data collection (i.e. Albanian, Romanian, Russian, Macedonian).

The interview format will follow a “computer-assisted self-interviewing” format (CASI), in which the interviewer will read out the questions whilst the participants select their answers on the tablet, with support where needed. For sensitive questions referring to intimate partner violence, child maltreatment, and parents’ history of abuse during childhood, we will employ an audio-CASI interviewing method, which allows participants to listen to the recorded question and answer privately on the tablet. Alternatively, parents can read the question and answer the items by themselves on the tablet.

If restrictions due to the COVID-19 pandemic will not allow in-person assessments, interviews will be completed over the phone. Because parents will not have the option to answer items privately over the phone, the sensitive items will not be administered during phone assessments. If restrictions due to the COVID-19 pandemic do not allow in-person assessments at post-test and follow-up assessment, parts of the assessment will also be offered online (the sensitive items) so that parents may answer the sensitive items privately via the online survey. If sensitive baseline measures (parents’ own history of child maltreatment) cannot be administered during the pre-assessment (due to phone assessment mode), they will be assessed at the next possible assessment point (e.g. post-assessment).

Approximately 4 months after the pre-assessment, post-test assessments will be carried out with the help of the same research assistants, blinded to the study conditions (planned for May / June 2021). Around 12 months after pre-assessments, we plan to carry out the follow-up assessment (December 2021–February 2022).

**Fig. 1:**
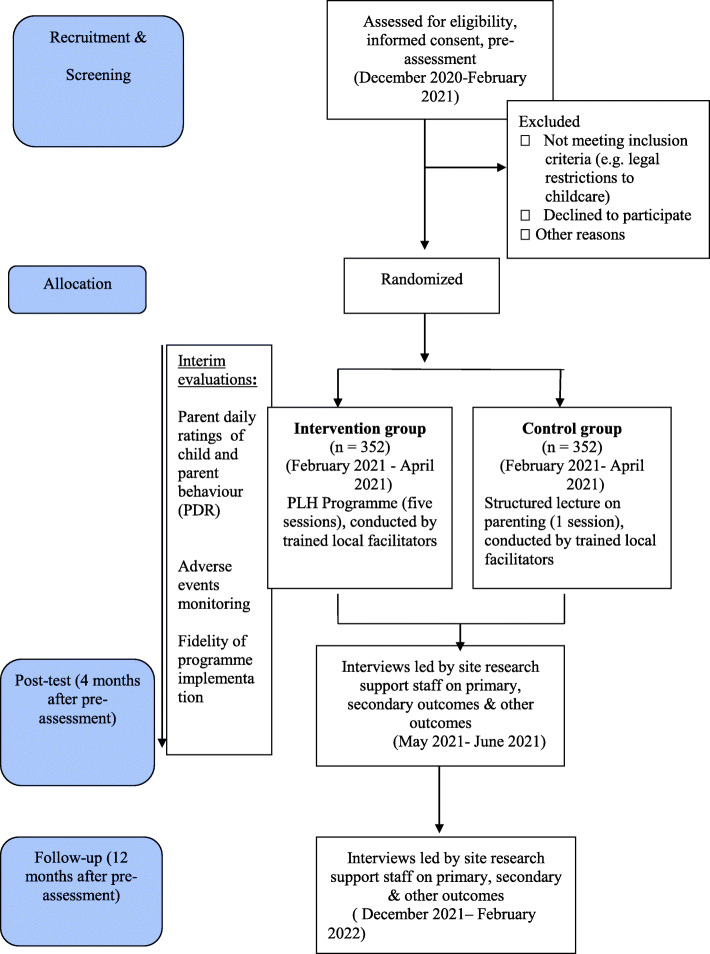
Study flowchart

Three repeated ratings of child and parent behaviour in the past 24 h will be assessed over the phone between pre-intervention and post-assessment. For the PLH-YC group, after the 1st, 3rd, and 5th session (lecture: after the lecture, 2 weeks and 4 weeks later), research staff will phone participants in order to monitor the adverse events and to assess parent and child behaviour, see Fig. 1Appendix 2—Study flowchart, for a tentative timeline and Additional file 1—Schedule of enrolment, interventions and assessments (SPIRIT Figure).

#### Interventions

##### Control group

Parents allocated to the active control arm will receive a structured power-point presentation on parenting and child development issues, called “Raising Healthy Children” (duration: 1 to 1.5 hours). The structure and contents were developed by University of Bremen and reviewed by the project partners, and the material is available upon request. Four topics will be covered: (1) Stages of child development; (2) Potential risk factors for child internalising problems; (3) Resources and protective factors; (4) Tips: What parents can do to promote children’s development. The choice of this comparator was made so as to ensure that parents from the active control benefit from useful information in dealing with their children (given that all recruited participants reported having a child with elevated levels of behavioural difficulties will have children with behavioural problems). At the same time, when conceiving the lecture, we made sure we minimised the effects of potential confounds in the control group that would make it similar to the intervention, such as details on parenting strategies, programme length, non-didactic approach (lecture in this group versus collaborative work in the intervention group), and hands-on activities (no home activities for this group).

Facilitators delivering this lecture will have the same/similar expertise to that of the facilitators engaged in PLH. Facilitators will participate in a brief training explaining the purpose of the lecture, clarifying the contents and addressing potential concerns. They will also be trained to follow the protocol to minimise risk of contamination between the two conditions (e.g. only allow minimal discussion within the lecture, answer questions in a didactic way).

##### Intervention group

The optimised version of PLH-YC will be delivered over five weekly sessions using a participatory, non-didactic approach to engage parents in learning positive parenting and child behaviour management skills. Programme activities include illustrated comics modelling how to implement key parenting skills, home activity assignments to apply these skills with their children, and group discussions addressing challenges experienced when applying home activities. The programme also includes simple mindfulness stress reduction exercises such as “Taking a Pause” to help parents cope with stress and reactivity towards their children. There is a facilitator manual for each session, and parents receive a PLH parent book (long versions in English, Macedonian, and Romanian available here: https://rise-plh.eu/work-packages/work-package-2/). For the current study, the facilitator manuals and parent handbooks of the optimised intervention will also be translated into Albanian and Russian. If the programme is delivered in person according to the standard protocol, each PLH-YC group with 12 parents will be delivered by two facilitators. Locally recruited professionals and non-professionals (e.g. teachers, psychologists, social workers, and peer parents) with prior experience delivering the programme during Phase 2 of the RISE project will serve as facilitators. All facilitators participated in a 40-h standardised training prior to delivering the programme for the first time. They will also receive an additional 16-h booster training prior to the RCT.

Programme and lecture delivery will start as soon as the programme implementers can organise a group of approximately 12 participants per condition who have completed baseline assessments. Facilitators of both groups will receive supervision on demand by trained coaches who have also previously provided coaching to facilitators during Phase 2.

##### Intervention contingency plan in the context of SARS-CoV2 pandemic

All three countries face significant challenges in dealing with the COVID-19 pandemic, challenges that might seriously interfere with the trial implementation. The stepwise safety plan is based on local restrictions and the CORONA traffic light system (https://vis.csh.ac.at/corona-traffic-light/world/) and includes (a) applying safety measures to reduce risk of infection during in-person meetings (e.g. keep distance, wear masks); (b) reduce the PLH parenting group size to six parents and one facilitator per in-person group (instead of 12 parents and two facilitators per group); and/or (c) switching to online video-conference delivery in areas where in-person delivery is no longer possible (PLH-YC: six parents per group + one facilitator, lecture: 12 parents per group + one facilitator). In these situations, we will deliver the optimised PLH programme and the lecture online via video-conferencing technology (e.g. Zoom, Microsoft Teams, or another secure platform in compliance with the GDPR and ethical guidelines for data safety). Facilitators of both conditions will receive a brief training on how to conduct the groups via online meeting software.

### Compensation of participants

Caregivers will receive a voucher (8–20€) and a snack (in-person assessment only) per assessment. The value varies between countries, because the buying power of a preset amount of money is different across countries. Also, if parents participate in all three phone calls (daily ratings between pre- and post-assessment), they will receive a gift. Any second caregiver participating in assessments will also receive compensation. This will be smaller given that the assessment is much shorter.

Parents from both groups will receive a snack (or a food voucher for the same amount), childcare, transportation support (if needed) for each session when delivered in-person, and a certificate of completion at the end. In the PLH-YC intervention group, parents participating in at least four out of five sessions will get a small gift. If interventions are carried out online because of the restrictions imposed by the COVID-19 pandemic, participants will receive internet cards or another voucher of the same amount to ensure that barriers for participation (i.e. connectivity issues) will be removed.

### Study outcomes

#### Primary outcome

*Child aggressive behaviour* will be assessed with the respective subscale of the parent-report Child Behavior Checklist (CBCL) 1½–5 and 6–18 years old [43]. Parents rate the occurrence of certain behaviours of their child on a 3-point Likert Scale (0 = *not true* to 2 = *very true* or *often true*).

*Child externalising disorders* will be evaluated by using The Mini International Neuropsychiatric Interview for Children and Adolescents – Parent Version (MINI-KID-P) [44]. The MINI-KID-P will be used to assess whether the criteria (based on ICD-10 and DSM-5) for (a) Conduct Disorder (CD) (F91.1, F91.2, F91.9) or (b) Oppositional Defiant Disorder (ODD) (F91.3) are met (yes/no). The results of the two disorders will be combined to one binary total score with 0 = *no externalising disorder* and 1 = *current externalising disorder* (ODD or CD).

On three occasions between baseline and post-test assessments, Parent Daily Ratings (PDR) [45] will be used to monitor *child oppositional and aggressive behaviour problems* that occurred within the last 24 h (answer format: *did occur/did not occur*). In order to minimise drop-outs, we will only administer the oppositional and aggressive subscale (10 items + 2 positive items) instead of the full PDR. We excluded the last item from the subscale (“he/she pouts”) because this question caused translation problems in the three implementation countries. The item was not understood correctly by parents and assessors and thus did not result in valid answers. The PDR will be assessed over the phone three times between pre- and post-assessment.

#### Secondary outcomes

*Child internalising problems* will be evaluated by using the internalising subscale of the parent-report versions of the CBCL 1½–5 and 6–18 years old [43]. The internalising subscale raw scores range from 0 to 62 (CBCL 1½–5 version; 31 items) and 0 to 64 (CBCL 6–18 version; 32 items) with higher scores indicating more problems.

*Parenting behaviours* will be assessed in four ways. The Laxness and Over-reactivity Subscales of the Parenting Scale will be used, measuring dysfunctional parenting practices [46]. Second, we apply self-reported measures of positive parenting and effective discipline (Parenting of Young Children Scale) [47]. Third, we will add five items from the Alabama Parenting Questionnaire (APQ) phone interview (three items for positive and two for negative parental behaviours) [48, 49] to the PDR phone assessment for assessing daily reports of effective parenting behaviour. The APQ answer format was adapted to fit the PDR format (*did occur/did not occur*). Fourth, child maltreatment will be assessed using the ISPCAN-Child Abuse Screening Tool – Trial-Children (ICAST-TC) [50].

For *parent-child relationship*, the Five-Minute Speech Sample (FMSS) [51, 52] will offer insight into caregivers’ attitudes and feelings about their child and the quality of the relationship. The parent is instructed to talk about his/her child for 5 min. The parent’s response will be audio recorded. The parent-report is then rated by trained coders, e.g. with regard to coherence, as an indicator for parent-child relationship [53]. We will also use the subscales Warmth and Criticism of the Family Affective Attitude Rating Scale (FAARS, [54]).

*Parental mental health* measures will tap into depression, anxiety, and stress symptoms by using the eponymous DASS-21 [55]. Three subscales can be derived, measuring caregiver symptoms of stress, anxiety, and depression (seven items each subscale). Total DASS scores range from 0 to 63, with subscales from 0 to 21.

*Parenting stress* will be measured using the Parenting Stress Scale (18 items, [56]). *Parental relationship measures* will include assessments of intimate partner violence and relationship quality. Intimate partner violence (physical and psychological victimisation and perpetration) will be evaluated with a screening instrument—family maltreatment measure [57] and adapted short form of the Revised Conflict Tactics Scale (CTS2S) comprising 29 items [58]. Couple satisfaction will be assessed using the Couple Satisfaction Index (4 items) [59].

*Child quality of life* will be measured using the Child Health Utility 9D (CHU9D; nine items). The sum score of the CHU9D ranges from 9 to 45, with higher scores indicating lower levels of quality of life.

#### Implementation outcomes

*RE-AIM Reach measurements* will comprise enrolment rate (total number of caregivers who attend the first session of the PLH/ the lecture group divided by the number of families recruited in that condition) and, for the PLH groups, the participation rate (percentage of the five sessions attended).

With regard to *RE-AIM implementation quality*, for both groups, programme implementation will be assessed in terms of implementation fidelity and adherence. The number of total activities actually implemented (yes/no), divided by the number of activities by facilitator (assessed via a facilitator checklist) required in the PLH-YC programme manual/lecture content, will be used as an indicator of *implementation fidelity.* Fidelity assessments will be self-reported by facilitators as well as assessed by external raters during live coded sessions (see below).

For *quality of delivery*, *competent adherence* of PLH facilitators to the programme activities and their delivery skills will be assessed with the PLH-Facilitator Assessment Tool (PLH-FAT) [32]. Trained assessors will attend one to two sessions to do live codings using the PLH-FAT. Seven distinct behavioural categories will be grouped into two scales, describing core activities and process skills. The assessment of core activities comprises modelling skills, collaborative work between facilitators, engaging participants, and leadership skills. Coaches assessing facilitators will not be blind to allocation. The delivery skills of the lecture facilitators will be assessed with one overall quality of delivery item (0 = inadequate, 4 = excellent), which will be rated by trained coders.

#### Cost outcomes

All persons involved in programme design, implementation, participation (as a beneficiary), and evaluation will complete cost diary sheets, detailing all the financial (and/or time) costs associated with each activity that they perform. Costs will be divided into the following two components based on the processes necessary to set up and deliver the programme: [1] set-up costs (e.g. initial training costs, and set-up before the start of the programme) and [2] programme delivery costs (e.g. travel to group sessions, room preparation, running the group sessions, room rental for programme delivery, administrative costs, and materials/supplies). Details of costs recorded previously for the cost-effectiveness analysis were presented elsewhere [32]. In addition to these, we will estimate the costs needed to access other social and mental health services available in each implementation site (e.g. by assessing participant use of other mental health services or helpline counselling services for child or parent mental health issues; as well as child emergency room and child welfare services).

#### Other outcomes and exploratory measurements

##### Other pre-specified outcomes

*Parental general health* will be evaluated with three items from the Medical Outcomes Study (MOS) Short Form-12 Health Survey [60]. The items capture potential difficulties in daily activities because of health problems, parent’s overall mental health, and physical or mental disabilities of the parent and/or the child.

For *change in prevalence of ADHD*, The MINI-KID-P (structured clinical interview, parent-report version) will be employed to assess whether or not the criteria for ADHD (F90.0, F90.1, F90.2) are currently met (yes/no). The results will be combined into one binary total score 0 (no ADHD) and 1 (current ADHD, criteria met).

*Interparental conflict* will be evaluated using the O’Learly Porter Scale (ten items) [61]. *Coparenting quality* will be assessed with two subscales (Agreement and Undermining) of the Coparenting Relationship Scale [62]. *Parental Self-Regulation* will be assessed using The Pause item assessing adult self-reported ability to pause before reacting reflexively to negative child behaviours. This assessment measures the frequency of parents taking a moment to think or calm down before reacting when he or she feels upset or stressed with the child. *Frequency and quality of family dinner* will be assessed using the Family Dinner Scale (unpublished measure developed by Anne Fishel).

*Social support* will be assessed with the Medical Outcomes Study (MOS) Social Support Survey – Emotional Support Subscale (eight items). Parents report on how often they receive emotional support on a Likert scale (1 = none of the time; 5 = all of the time) [63]. These will be complemented by self-report of *alcohol misuse* using three items from the Alcohol Use Disorders Identification Test (AUDIT-C) [64].

*Parent, child, and family demographic* data will be collected with UNICEF Multiple Indicators Cluster Survey (MICS) Household Survey [65]. MICS includes assessments of basic literacy, child’s relationship to the caregiver, presence of child’s biological parents (including reasons for absence), and other household members’ age, gender, and relationship to the caregiver.

*Family poverty* will be assessed with three measures. One measure assesses household assets (numbers of mobile phones and internet access) and is a modified five-item version of the MICS [65]. The current items were included based on previous analyses in each of the three countries that demonstrate what household assets are appropriate to assess (significant variance, accessibility). We will assess participants’ income after covering for the basic expenses with one item, with options ranging from *1 = enough to that I/we can comfortably purchase most of the things we really want* to *3 = not enough to purchase much of anything I/we really want* (i.e. after paying for essential expenses like food, housing, utilities, child care, and medical care). *Family hunger* will be assessed with the Food Insecurity Experience Scale [66]. The FIES consists of eight items with a dichotomous response pattern (“*yes*”/”*no*”) and a “*don’t know*” option [66].

*Impact of COVID-19 pandemic* will be assessed with three ordinal items targeting (a) the amount of stress felt by the parent because of COVID-19; (b) the extent of the (potential) negative impact that the pandemic has had on the child; (c) the extent of the (potential) negative impact of COVID-19 on the family.

*Parents’ exposure to adversity* and *maltreatment* during the first 18 years of life will be assessed with the Adverse Childhood Experiences Questionnaire (ACE-Q), a ten-item scale (each item with a “*yes*” or “*no*” response option) tapping into emotional and physical abuse, physical neglect, and abuse related to atypical households [67]. A modified version of the Child Abuse Screening Tools Retrospective version (ICAST-R) [68] will comprise three items to assess the history of physical maltreatment and verbal abuse.

There will be a short version for the assessment of the secondary caregivers. This comprises the CABI, parenting stress, positive parenting, parental couple satisfaction, and alcohol misuse.

### Data management and data analysis plan

#### Data management plan

The University of Klagenfurt is responsible for monitoring data collection, ensuring data quality, planning statistical analyses, and keeping the rest of the steering committee informed on the progress of these activities.

Survey and audio data collected electronically will be anonymised (participants are identified through numerical codes) and uploaded weekly to a central secure data server at Klagenfurt University. Access to the data will be granted only to the members of the research team. Electronic equipment used for data collection (tablets, audio-recorders) will be password-protected and kept in a locked cabinet when not in use. Paper versions of questionnaires, adverse events forms, informed consent sheets, identification sheets and other hard copy documents used in data collection will be archived by researchers and stored safely by the local teams in the country of the data collection. De-identified adverse event forms will be shared with ethical oversight teams from the Universities of Bremen and Klagenfurt. All electronic and paper data will be kept by each site for ten years, in accordance with the Code of Conduct at the University of Klagenfurt (https://www.aau.at/en/research/research-profile/good-academic-practice/).

#### Data analysis

##### Sample size calculation

To allow detection of small effect sizes, we will estimate sample size based on a *d* = .25 (*f* = .10) for the primary outcomes. Detecting this effect in a repeated measures ANOVA at an alpha of .05 whilst ensuring a power of 80% requires a total sample of *N* = 704. We will examine the change in the primary outcomes across three time points (pre, post, and follow-up) within the overall sample using latent growth modelling and conduct a multi-group analysis to test differences across countries. To reach the required sample size of 704, we will recruit a total of 864 families for baseline assessment, assuming approximately 17% drop-out rate based on our previous phase of data collection [69].

##### Primary and secondary outcome analyses

Data will be analysed using an intention-to-treat approach, in line with the study protocol and with good practice in RCTs [70]. Missing data will be handled using either multiple imputation or Full Information Maximum Likelihood (FIML) method [71].

The primary analysis will consist of testing comparatively the magnitude of change in child aggresive behaviour, in the control vs. PLH-YC participants, at post-test and follow-up. Identical analyses will be run for the secondary outcomes (i.e. child internalising problems, dysfunctional parenting strategies, positive parenting, daily reports of effective parenting behaviour, child maltreatment, parental mental health, IPV, couple satisfaction, parenting stress, quality of parent-child relationship, and child ’s quality of life). The main analyses will involve testing pre-post or pre-follow-up differences controlling for pre-intervention levels whilst accounting for covariates. Maximum likelihood estimation with robust statistics will be used with Mplus 8.2 software to account for non-normal distributions in measures and also apply a FIML framework for handling missing data at post- or follow-up. The models will be further tested using latent growth curve models adjusting for country and pre-assessment levels of the outcome variables (e.g. age as a time-invariant covariate). Indirect effect models will be tested in a structural equation framework and include participation rate and fidelity as mediators for the PLH-YC group and enrolment rate as the mediator across groups. In additional analyses, we will also model the effects of participation (i.e., attendance). This will be analysed with Complier Average Cause Effect models that estimate the magnitude of the paths based on the baseline scores in predictors (e.g. family adversity) and actual participation [72]. Further clustering will be within groups (facilitators) and included in subsequent analyses as needed. The analyses for MINI-KID ODD/CD positive screen will be conducted with logistical regression models due to the categorical nature of the data. Depending on the distributions and on other descriptive indicators, Bayesian analyses will be used as deemed appropriate.

##### Other subgroup analyses

To the extent to which we will have enough secondary caregivers (e.g. fathers) in the programme, it will be of interest to test whether parent gender acts as a moderator of programme effects on child reported behavioural and emotional problems. Child gender will also be included as a covariate in the models of outcome, testing both main effects and interactions between covariates and intervention.

Other subgroup analyses will explore, across and within countries, the role of different cultural, economic, and societal factors on the pre-specified outcomes. Particular attention will be given to vulnerable populations (ethnic minorities and/or economically disadvantaged groups) and whether intervention effects on outcomes will be similar to those for non-disadvantaged individuals. For instance, we aim to test the moderating role of parental (mental) health, ethnicity, poverty, and parenting practices (harsh parenting) on primary and secondary outcomes. Given the increased power required for moderation analyses, these analyses will be considered exploratory.

##### Cost-effectiveness analysis

The objective of this analysis is to evaluate the costs and cost-effectiveness of the PLH parenting programme vs. the lecture on parenting. Cost-effectiveness ratio will be calculated in terms of Euros per one point reduction of the CBCL aggressive subscale score in the PLH-YC programme. Besides the use of CBCL aggressive subscale score as the primary outcome for the cost-effectiveness analysis, other outcomes such as Parenting Scale (PS), Parenting of Young Children Scale (PARYC), and Quality of life (Child Health Utility 9D) will also be used to better inform decision making.

We will conduct cost analyses based on cost diaries and on cost estimations for participant access to alternative social/mental health services in each site. Programme costs will be calculated using a micro-costing approach, multiplying resource use by unit costs. Our micro-costing will be conducted from the payer’s perspective, excluding participants’ out-of-pocket costs and the opportunity costs associated with participant time. We are interested in the cost categories and the extent to which they vary across sites and subgroups. Cost-effectiveness ratio (outcomes expressed as natural health outcome units) and cost-utility ratio (outcomes expressed as quality-adjusted life years) will be calculated to assess and compare the cost-effectiveness of PLH-YC across sites and populations. Depending on data availability, either extended cost-effectiveness analysis (ECEA) or distributional cost-effectiveness analysis (DCEA) will be further conducted to incorporate equity into an economic evaluation of PLH-YC. ECEA provides breakdowns of the costs and outcomes of health interventions by social group. DCEA, in addition, provides a summary measure of equity impact and analyses the potential trade-offs between equity and efficiency impacts. Furthermore, based on the cost-effectiveness results, the resources required to provide the optimal PLH programme on a large scale will be estimated for North Macedonia, the Republic of Moldova, and Romania respectively, and barriers to broader implementation will be discussed.

##### Additional exploratory analyses

We plan to assess service use of participating families (e.g. other mental health services, emergency room visits, child welfare services) at post- and follow-up assessments. However, because of the low base rate of service delivery in the three countries, we do not expect change in these indicators, but rather these will be used as part of cost analyses. Additional exploratory analyses will comprise subscales of the FMSS: If inter-rater reliability of subscales is adequate (ICC of .70 or higher), the subscales concern (1 “no worry and concern” to 7 “thematic concern and worry”), acceptance (1 “strong rejection” to 7 “high warmth and acceptance”), and separateness (1 “no clear separation” to 7 “complete separateness”; incl. boundary dissolution (BD) answer format: 0, 1, 2 with higher scores indicating more BD) will be analysed.

### Ethical considerations and dissemination plan

This protocol is in full compliance with institutional and international regulations regarding human rights (Declaration of Helsinki). Prior to enrolment, all caregivers, facilitators involved in data collection and intervention delivery will receive information letters and give their informed consent to take part. Where written consent during in-person assessments is not feasible (due to COVID-19 restrictions), we will obtain oral consent from parents (over the phone). They will be informed about their right to withdraw at any time, without suffering any negative consequences, as well as about their right to address enquiries or complaints to the local or central ethical boards.

#### Adverse event assessment procedure

Information about adverse events (AE) will be collected throughout trial implementation (at pre-test and at follow-up, as well as during the three phone assessments between pre-and post-assessment at the same time as administering the PDR). With a standardised checklist (12 items), newly occurred or worsened physical/medical problems (three items; e.g. accident), behavioural problems (three items; e.g. aggressive/violent behaviours), emotional problems (one item), and other significant problems in daily life (five items, e.g. unplanned hospitalisation) will be collected as well as the subjective ratings of their severity (1 = mild, 4 = severe; answer format significant problems in daily life: happened: yes/no). If an AE with a severity of ≥ 3 or a significant problem in daily life is reported, a follow-up questionnaire will be completed in order to get a better understanding of what happened (e.g. detailed description of the event and actions taken, outcome, happened to child/caregiver). Based on the participant’s report, the research teams will aim to classify the magnitude of the AE as well as the likelihood that it is related to the project. Serious adverse events (SAE) will be reported to the local ethical boards as well as to the Data Safety and Monitoring Board (DSMB) and the central institutional review board (IRB) in Klagenfurt. The DSMB is independent from the consortium or the funding body and comprises two senior researchers with specialisation in parenting programmes, child development and child mental health who regularly review study protocol amendments, data collection and implementation procedures, participant safety, and study ethical aspects. The DSMB is notified immediately with regard to SAE occurrence, study misconduct, or other breaches in ethics and is responsible for the decision to continue, amend, or stop the trial immediately.

#### Dissemination of trial results

Community-level dissemination strategies will involve local and national key stakeholders in each of the implementing countries, who will periodically receive written briefs about the status of the project and will take part in stakeholder meetings. Schools, kindergartens, teachers, and parents will be informed through previously established channels: information and thank you notes, social media channels, etc. The project website (http://www.rise-plh.eu) is regularly updated with research results. Publications emerging from the trial will be reported in open-access journals or open-access repositories, and metadata and anonymised data on the aggregate level will be uploaded on Zenodo after the publication of the main study results (https://zenodo.org/).

## Discussion

This is the first study, to our knowledge, that will implement a multi-site RCT to test a standardised, optimised, and promising parenting programme in LMICs. The results will allow insights into the efficacy of PLH-YC in alleviating child behavioural and emotional problems, programme cost-effectiveness, its transportability in three different cultural contexts, and its potential for scalability. Having an efficacious and cost-effective parenting intervention that is easy to implement in disadvantaged socio-economic contexts, in times when child mental health problems are increasing [73, 74], will be an important step forward towards keeping the spotlight on child mental health issues in Southeastern Europe. The knowledge can be used to transfer PLH-YC to other LMIC and thus create access to affordable, evidence-based services.

### Trial status

Protocol version 01 for the RCT (Phase 3). Recruitment started on December 7, 2020, and was completed on May 17, 2021. The programme delivery was completed on July 13, 2021, and the post-assessments on September 24, 2021. For details, please refer to the trial registry (NCT04721730). This will be updated on a regular basis.

### Supplementary Information

**Additional file 1.** Schedule of enrolment, interventions and assessments (SPIRIT Figure).

## Data Availability

Data will be shared among the RISE research team. It is planned to make some anonymised datasets available to the public and other researchers via an open-access repository following the FAIR (Findable, Accessible, Interoperable, Reusable) principles. The research team will ensure that results will be published in open-access peer-reviewed journals.

## Appendix

### Appendix 1. Information sheet and consent form for parents and caregivers

**Institutions:** Babes Boylai University, Cluj-Napoca (Rumania); Institute for Marriage, Family and Systemic Practice – ALTERNATIVA (North Macedonia); Health for Youth Association (Republic of Moldova); University of Bremen (Germany); Alpen-Adria-University Klagenfurt (Austria); Bangor University, Wales (United Kingdom); University of Cape Town (South Africa); Georgia State University (USA); University of Oxford (United Kingdom).

**Sponsor:** This project has received funding from the European Union’s Horizon 2020 research and innovation programme under grant agreement No 779318.

**Ethics Approval:** by the Human Research Ethics Committee of the Alpen-Adria-University Klagenfurt and the *[local institutions of the respective country]*

**RISE**

**Information Sheet for Parents**

- **We are asking you to be in a research study.**
- **You do not have to be in the study.**
- **If you say yes, you can quit the study at any time.**
- **Please take as much time as you need to make your choice.**

**Why am I asked to be part in this research study?**

We want to learn more about how well two support services for families work: one is a parenting programme called *Parenting for Lifelong Health for Young Children, PLH Children*. The other is a lecture for parents called *Raising Healthy Children*.

We are asking people like you who have a child aged 2 to 9 years to help us. A total of 864 parents will be part of the study, 288 in [Country].

**What if I don’t understand something?**

- This form may have words you don’t understand. Research staff will read it with you, if you like.
- You may ask as many questions as you like before you decide whether you want to be in this study.
- You are free to ask questions at any time before, during, or after you are in the study.

**What if I say yes, I want to be in this study?**

We first will see if you fit into the study. Therefore, we will ask some questions about your child.

To be part of the study, you need to be a parent or caregiver of a child between the ages of 2 and 9 years whose behaviour you are having challenges with. You also have to agree to participate in the parenting programme, provide consent in the full study, and have language skills to participate in a parenting programme.

If you qualify, we will do these things

- Ask about your life, your feelings and your relationship with your child
- Read the questions out loud and enter your answers in this electronic tablet
- Let you listen to questions by an audio record
- Give you a brief form with questions about adverse events
- Let you skip any question you do not want to answer
- Two sections of the interview will be audio recorded (one for all parents and one only for parents with a child aged 6 years or older). If you do not want to be audio recorded, please tell us and we will not record these sections.

This will take about 75 min.

There are two support services (the parent grogram/the lecture) which might be helpful. In order to have a closer look what works best with families like yours, there will be a randomisation process. Randomisation means that we will put you into one of the activities (the parenting programme or the lecture) by chance. Importantly, you get some support, no matter to which group you are assigned.

- Both services will take place in groups with other families.
- If the groups are in person, your child will not attend the groups but childcare will be provided if you need it in order to attend the group.
- The parenting programme (5 sessions):
  - The programme wants to improve relationships between parents and children. Parents also learn strategies how to deal with their children in challenging situations.
  - Two people will deliver the programme in community centres / clinics.
  - Before, you will have an individual meeting with your group leader. This person will explain the programme in detail to you. During the parent groups, you will do activities and also practice at home. You will only have to do the activities if you wish to.
- The lecture on parenting (1 session):
  - The lecture wants to inform parents about the stages of child development and potential risk and protective factors for child mental health problems. The leader will also share tips for parents to support the healthy development of children.
  - One person will deliver the lecture in community centers / clinics.
- Someone from the research team will observe the session(s) in order to see how well the facilitator is delivering the group. Everything that is said during the session will be kept private.
- We want to know how you and your child are doing between the first and the second interview. We will call you over the phone 3 times after the first interview. Each phone call will take about 10 minutes and we will ask you questions about the wellbeing of you and your child.
- We will contact you again after completion of the programme/the lecture. We will ask you the same questions that we will ask you at the beginning. This will take about 75 minutes.
- We will then contact you about 6 months later to ask you the same questions for the last time (approximately 60 minutes). All the interviews will take place at a community center / university clinic, you can decide.
- If you are currently in a relationship, we will also invite your partner/spouse to participate in the study, but they are not required to participate for you to be part of this study.

**COVID-related precautions**

If we cannot meet in-person because of the restrictions due to COVID-19 pandemic, this will change:

- The interviews will be done over the phone. You will be asked to answer some questions during the second and third interview over the phone or online via a private weblink.
  - During the second and third interview, the study research team will ask for your email address or phone number to send the weblink. Information that could identify you will be stored separately from any of your responses during the interviews/surveys (Limesurvey or ODK).
- The parent groups will be online using a secure online platform (e.g. Zoom, Microsoft Team, Classroom). Only the facilitators and the other parents can join the online meeting (with a link and a password). During the online groups, we cannot offer childcare.
- Please be aware that the online platform that is used has their own data protection procedures that are available on the platform’s website.
- Please let us know, if you would not participate if the groups need to be online.

Based on the local situation in *[Country]* now, we will start with online groups.

**What if I say no, I do not want to be in this study**?

▪ Nothing bad will happen.

**What happens if I say yes, but change my mind later?**

▪ You can stop being in the study at any time.

▪ Nothing bad will happen.

▪ You do not have to give any reasons.

▪ If you wish to be taken out of the study, please contact *[Country PI]*

**Who will see the information about me that is collected?**

- We will store all of your research records in locked cabinets and secure computer files. Only the research team has access. We will take your name off of any information where this is possible.
- Personal identifying information needed for research purposes will be kept for 10 years, after which it will be destroyed. Identifying information such as your name and contact details be destroyed at the end of the study unless you agreed to be contacted in the future in which case we will only keep your name and contact details. At any point during the study, you can withdrawal your participation and request that personal identifying information be deleted.
- If an online platform is used due to COVID-19 precautions, the platform (e.g. Zoom, Microsoft Team, Classroom) has their own data protection policies for collecting and storing data for any visitors to their platform. Please review their company policies on data protection for more information.
- We will keep all your anonymized as well as personal information confidential as provided by law. The only exception is any risk of possible harm to you or others. If a child is harmed or is at risk for harm, the research team will consult with one another and decide on the best course of action in line with international UNICEF Child protection standards and the Child Protection standards and Policies in your country.
- We will share our study results via the Internet and an open database. Your name or address or other personal identifying information will not appear.
- We will share the results of the study in academic journals, research reports and at conferences. We will take off your name or any other identifying information.
- After the study is finished, you can see the results of the study on our website, www.rise-plh.eu.

**Will it cost me anything to be in the study?**

The study will not cost you anything.

**Will I be paid?**

You will be receiving a food/gift voucher (approximately 10€) after the end of each interview. Also, you will receive a gift if you participate in all phone call interviews.

If the groups are in-person, during the parent programme/ the lecture you will receive a snack and a transport voucher if necessary.

If you are assigned to participate the parent programme and attend at least 4 out of 5 session, you will get a small gift.

If the groups have to be online due to COVID-19, you will receive a pre-paid data voucher or equivalent [COUNTRY INSERT AMOUNT) when you learn about which group you are assigned to instead of the snack and transport voucher.

**Will being in this study help me in any way**?

- You will be able to participate in the lecture or the parenting programme for free.
- Being in the study may or may not help you, but may help other parents to have a better relationship with their child in the future.
- We do not know whether being in the study and the programme activities specifically will help you individually but we do know that the programme activities have helped many other parents like you throughout the world. The lecture informs parents about the stages in development of children. It may help to better understand your child and if his/her behaviour is in the normal range.

**What are the risks of being in this study?**

- The risks of this study are no more than what happens in everyday life.
- The questions we will ask may make you feel sad, upset or uncomfortable. In that case, we can refer you to support services.

**What if I have questions**?

✓ Please call the local head researcher of the study [*name of local coordinator]* if have any questions about this study

✓ Have questions about your rights

✓ Feel you have been injured in any way by being in this study

✓ Have questions about this study

✓ Have questions about your rights

✓ Can’t reach the study team

✓ Need to speak to someone not directly involved with this study

**What should I do if I want to be in the study**?

✓ Sign this form

✓ You can wait up to 7 days to decide whether you want to be in the study or not.

We will give you a copy of this informed consent form to keep.

**Consent Form for Parents in the Randomised Controlled Trial**

**By agreeing to the project, I am saying that:**

- I understand that joining this study is voluntary.
- I agree to be in the study.
- Someone talked with me about the information in this document and answered all my questions.
- I understand that the information I provide (without any identifying information) may be combined with other families’ experiences of similar programmes from other countries so that we can understand how they work across the world.
- For online meetings: I am responsible for the security on my computer. I understand that I may not share the link and the password for the online meetings with other persons.

**I know that:**

- I can stop any and all parts of the study at any time and nothing bad will happen to me.
- I can contact the chair *[Phone/ email/institutional address of the Chair of the Local Ethical Committee]* of if I have any questions about the study or about my rights.
- I do not give up any of my rights by signing this form.

Date:

□ Yes, I agree

□ No, I do not agree

### Appendix 2

.

## Abbreviations

ACE-Q: Adverse Childhood Experiences Questionnaire
ADHD: Attention deficit with hyperactivity disorder
AE: Adverse events
ANOVA: Analysis of variance
APQ: Alabama Parenting Questionnaire
AUDIT: Alcohol Use Disorders Identification Test
BD: Boundary dissolution
CABI: Child and Adolescent Behavioral Inventory
CASI: Computer-assisted self-interviewing format
CBCL: Child Behavior Checklist
CD: Conduct disorder
CHT9D: Child Health Utility 9D
CTS2S: Revised Conflict Tactics Scale
DASS: Depression Anxiety Stress Scale
DCEA: Distributional Cost-effectiveness Analysis
DSMB: Data Monitoring and Safety Board
ECEA: Extended Cost-Effectiveness Analysis
FAIR: Findable, Accessible, Interoperable, Reusable
FDS: Family Dinner Scale
FIES: Food Insecurity Experience Scale
FIML: Full Information Maximum Likelihood
FMSS: Five-Minute Speech Sample
GDPR: General Data Protection Regulation
HIC: High-income countries
IRB: Institutional Review Board
ICC: Intraclass correlation
ICAST-R: Child Abuse Screening Tools Retrospective version
ICAST-I: Child Abuse Screening Tool-Intervention
IPV: Intimate Partner Violence
LMIC: Lower middle-income countries
MICS: Multiple Indicators Cluster Survey
MINI-KID-P: Mini International Neuropsychiatric Interview-Parent Version
MOS: Medical Outcomes Study
MOST: Multiphase Optimisation Strategy
ODD: Oppositional defiant disorder
PARYC: Parenting of Young Children Scale
PDR: Parent Daily Ratings
PLH: Parenting for Lifelong Health
PLH-FAT: Parenting for Lifelong Health-Facilitator Assessment Tool
PLH-YC: Parenting for Lifelong Health for Young Children
PS: Parenting Scale
RCT: Randomised clinical trial
RE-AIM: Reach Effectiveness Adoption Implementation Model
RISE: Prevention of Child Mental Health Problems in Southeastern Europe - Adapt, Optimise, Test, and Extend Parenting for Lifelong Health
SAE: Serious adverse event
UNICEF: United Nation’s Children Fund
WHO: World Health Organization
