# Supplementary material for: Prevention of child mental health problems through parenting interventions in Southeastern Europe (RISE): study protocol for a multi-site randomised controlled trial
Source: Trials. 2021 Dec 27;22:960. doi: 10.1186/s13063-021-05817-1 (PMC8710933; doi:10.1186/s13063-021-05817-1)
Supplement: Supplementary file 1 — Additional file 1. Schedule of enrolment, interventions and assessments (SPIRIT Figure). [file 13063_2021_5817_MOESM1_ESM.docx]

*APPENDIX C. Schedule of enrolment, interventions and assessments (SPIRIT Figure)*

|  |  | **STUDY PERIOD** | | | | | | | |
| --- | --- | --- | --- | --- | --- | --- | --- | --- | --- |
|  | **Enrolment** | **Allocation** | **Intervention** | | | | |  | **Follow-up** |
| **TIMEPOINT**** | ***T-1*** | **T0** | ***Session 1*** | ***Session 2*** | ***Session 3*** | ***Session 4*** | ***Session 5*** | ***Post-***  ***assessment (T -1 + 4 months)*** | ***T-1 +12 months*** |
| **ENROLMENT:** |  |  |  |  |  |  |  |  |  |
| Eligibility screen | x |  |  |  |  |  |  |  |  |
| Informed consent | x |  |  |  |  |  |  |  |  |
| Allocation |  | x |  |  |  |  |  |  |  |
| **INTERVENTIONS:** |  |  |  |  |  |  |  |  |  |
| Control (lecture “Raising Healthy Children” |  |  |  |  |  |  |  |  |  |
| Intervention (PLH YC) |  |  |  |  |  |  |  |  |  |
| **ASSESSMENTS:**  **Primary outcomes** |  | | | | | | | | |
| Child aggressive behavior | x |  |  |  |  |  |  | x | x |
| Prevalence of child externalizing disorders | x |  |  |  |  |  |  | x | x |
| Child oppositional and aggressive behavior (last 24h) |  |  | x |  | x |  | x |  |  |
| **Secondary outcomes** |  | | | | | | | | |
| Child internalizing behavior problems | x |  |  |  |  |  |  | x | x |
| Dysfunctional parenting | x |  |  |  |  |  |  | x | x |
| Positive parenting | x |  |  |  |  |  |  | x | x |
| Effective parenting behavior (last 24h) |  |  | x |  | x |  | x |  |  |
| Parent-child relationship quality: coherence | x |  |  |  |  |  |  | x | x |
| Parent-child relationship quality: FAARS | x |  |  |  |  |  |  | x | x |
| Frequency and incidence of child maltreatment* | x |  |  |  |  |  |  | x | x |
| Parent mental health | x |  |  |  |  |  |  | x | x |
| Parenting stress | x |  |  |  |  |  |  | x | x |
| Parental relationship quality | x |  |  |  |  |  |  | x | x |
| Intimate partner violence* | x |  |  |  |  |  |  | x | x |
| Child quality of life | x |  |  |  |  |  |  | x | x |
| **Implementation outcomes** |  | | | | | | | | |
| RE-AIM Reach: Enrollment rate |  |  | x |  |  |  |  |  |  |
| RE-AIM Reach: Participation rate** |  |  | x | x | x | x | x |  |  |
| RE-AIM Implementation: Fidelity lecture |  |  | x |  |  |  |  |  |  |
| RE-AIM Implementation: Fidelity PLH-YC |  |  | x | x | x | x | x |  |  |
| RE-AIM Implementation: Quality of delivery lecture |  |  | x |  |  |  |  |  |  |
| RE-AIM Implementation: quality of delivery PLH*** |  |  |  |  |  |  |  |  |  |
| **Cost outcomes** |  |  | x | x | x | x | x | x^a^ | x^a^ |
| **Other pre-specified outcomes** |  | | | | | | | | |
| Parents’ general health | x |  |  |  |  |  |  |  | x |
| Prevalence of ADHD | x |  |  |  |  |  |  | x | x |
| Interparental conflict | x |  |  |  |  |  |  | x | x |
| Coparenting quality | x |  |  |  |  |  |  | x | x |
| Parental Self-Regulation | x |  |  |  |  |  |  | x | x |
| Frequency and quality of family dinner | x |  |  |  |  |  |  |  | x |
| Social support | x |  |  |  |  |  |  |  | x |
| Alcohol misuse | x |  |  |  |  |  |  |  | x |
| **Other variables** |  | | | | | | | | |
| Parent, child and family demographic | x |  |  |  |  |  |  |  |  |
| Adverse events | x |  | x |  | x |  | x |  | x |
| Family poverty | x |  |  |  |  |  |  |  |  |
| Impact of COVID-19 pandemic | x |  |  |  |  |  |  |  | x |
| Parents’ exposure to adversity and maltreatment* | x |  |  |  |  |  |  | x |  |
| Parent’s history of physical and verbal abuse* | x |  |  |  |  |  |  | x |  |

Notes. *If sensitive baseline measures (parents’ own history of child maltreatment) cannot be administered during the pre-assessment (due to phone assessment mode), they will be assessed at the next possible assessment point (e.g., post-assessment). **Participation rate: only for caregivers allocated to the PLH condition. ***PLH: One to two sessions will be rated regarding quality of delivery. ^a^ Service utilization for cost analyses will be assessed at post and follow-up assessments.
